# Vascular and Neural Compression Syndromes Associated with Plantaris Muscle Variants: A Classification-Based Review

**DOI:** 10.3390/jcm15083006

**Published:** 2026-04-15

**Authors:** Łukasz Olewnik, Ingrid C. Landfald, Magdalena Łapot, Robert F. LaPrade

**Affiliations:** 1 Department of Clinical Anatomy, Mazovian Academy in Płock, 09-402 Płock, Poland; 2 VARIANTIS Research Laboratory, Department of Clinical Anatomy, Mazovian Academy in Płock, 09-402 Płock, Poland; 3 VARIA Research Laboratory, Mazovian Academy in Plock, 09-402 Płock, Poland; 4 Twin Cities Orthopaeedics, Edina&Eagan, Eagan, MN 55121, USA

**Keywords:** plantaris muscle, popliteal fossa, popliteal artery entrapment syndrome, tibial nerve, common peroneal nerve, MRI band sign, Duplex/Doppler, anatomical variation, risk stratification

## Abstract

**Background:** The plantaris muscle (PM) shows substantial variability in its proximal belly attachments. Although often deemed vestigial, specific variants may narrow or reshape the popliteal corridor and contribute to vascular (popliteal artery entrapment syndromes, PAES) and neural conflict (TN, CPN, sural nerves). Despite abundant anatomical descriptions of the plantaris, its contribution to neurovascular compression has not been organised into a classification-linked, imaging-integrated framework. **Objective:** To synthesise adult and foetal anatomical data with clinical–radiological evidence into a classification-linked framework that stratifies vascular and neural compression risk by proximal PM variants, and to propose an integrated risk matrix and variant-directed diagnostic/operative pathway. **Methods:** Narrative, classification-centred review centred on the Olewnik schema (Types I–VI) and multi-headed/accessory variants. We mapped variant geometry to (1) physiological compromise on provoked Doppler US and (2) anatomical correlates on MRI/MR angiography (MRA) (axial “band sign”), deriving graded risk for vascular and neural axes and an integrated, action-oriented grade per limb. **Results:** Baseline risk is low for canonical/compact footprints (Type I–IA, Type V), moderate for capsular-junction patterns (Types II/III), and potentially higher-risk for lateral linkage (Type IV; iliotibial band (ITB)/Kaplan fibres continuity) and multi-headed configurations (duplication, bifurcation, ≥3–4 heads; accessory proximal slips). The integrated matrix upgrades risk for a clear band sign, reproducible compromise on provoked Doppler US, or multi-headed/Type IV anatomy and downgrades when rigorous provocation is negative and muscle volume is small. We provide a variant-indexed imaging checklist, common pitfalls (e.g., Type IV misread as ITB thickening; multi-headed variants misread as cyst/tumour), and operative checkpoints to target capsular clefts, lateral bands, tunnels, and accessory slips. **Conclusions:** A classification-linked, imaging-integrated approach clarifies which proximal PM variants are plausibly associated with neurovascular entrapment (based on case-level evidence) and aligns work-up with targeted decompression and may improve diagnostic precision and inform surgical planning. **Clinical relevance:** The framework operationalises variant naming in reports, standardises dynamic provocation and axial mapping, and prioritises variants considered higher risk (Type IV; multi-headed) for early multidisciplinary review. Given that most clinical signals derive from case reports/series (Level IV), these recommendations are inferential and should be applied with clinical judgement.

## 1. Introduction

### 1.1. Background and Significance

The plantaris muscle (PM) is small yet highly variable; despite its long-standing reputation as vestigial, its clinical relevance is being increasingly recognised [1,2,3,4,5]. Variation encompasses proximal origin patterns (including supernumerary heads) and the course of the proximal tendon within the popliteal fossa, creating uncommon relationships with regional neurovascular structures [6,7,8,9,10,11,12,13,14,15,16,17].

### 1.2. Clinical Focus: Neurovascular Compression in the Popliteal Fossa

Specific proximal origin variants and the adjacent proximal tendon may contribute to popliteal artery entrapment syndrome (PAES) and to neural conflicts involving the tibial, common peroneal (CPN), or sural nerves (SN) [18,19,20]. Reports describe anatomical PAES attributable to aberrant PM (often in combination with the soleus) and rare proximally tendon-mediated contacts with the neurovascular bundle that can provoke neuropathic symptoms [8,19,20,21].

### 1.3. Knowledge Gap

Although PAES is well documented, there is no systematic mapping of discrete proximal origin morphologies (and the immediately adjacent proximal tendon segment) to graded neurovascular compression risk. Existing classifications (adult and foetal) have not been explicitly linked to a risk profile, despite accumulating case evidence that risk varies by variant rather than by the mere presence of PM [6,7,10,13,15,16,17]. While classical PAES taxonomies classify artery–myofascial relationships, they do not specify which proximal plantaris geometries are most likely to generate vascular or neural conflict; the present review addresses this gap with a classification-linked, imaging-integrated framework.

### 1.4. Aim and Scope

This review proposes a classification-based framework to stratify vascular (especially PAES) and neural compression risk according to proximal origin variants of the PM and the course of its proximal tendon in the popliteal fossa. We integrate adult and foetal anatomical data with clinical–radiological observations to perform the following:

1. Identify high-risk variants for PAES/neuropathy;
2. Present a practical risk table and recognition algorithm (clinical–imaging–intraoperative); and
3. Outline surgical implications and diagnostic pitfalls.

This review focuses on proximal-belly morphology; distal tendinous variants and insertional patterns are outside its scope.

### 1.5. Methods (Review Design and Evidence Level)

We conducted a narrative, classification-centred review to map proximal plantaris muscle variants to neurovascular risk strata. Searches were run in PubMed, Scopus, and Web of Science for English-language records published in 1990–2025. Core strings combined anatomy and compression terms were used, for example:

(“plantaris” or “musculus plantaris”) and (“popliteal artery entrapment” or “PAES” or “neurovascular compression” or “tibial nerve” or “common peroneal nerve” or “sural nerve” or “iliotibial band”) and (anatomy or MRI or ultrasound or duplex).

Inclusion criteria: Human studies (adult or foetal) that (1) describe proximal plantaris morphology (origin, number of heads/accessory slips, course) or (2) report imaging case evidence linking plantaris to popliteal vascular or neural compression, and (3) reviews/classification papers on PAES.

Exclusion criteria: Distal tendon-only variants; non-human studies; non-English texts; records without extractable proximal mapping; duplicates.

Study selection: Two reviewers independently screened titles/abstracts and assessed full texts; disagreements were resolved by consensus.

Data items: Variant type and neighbouring structures, imaging signs (e.g., band sign, dynamic provocation), clinical correlates, and management.

Evidence level: Most clinical inferences derive from case reports/series (Level IV); conclusions are therefore inferential and hypothesis-generating, not definitive. No protocol was registered and PRISMA reporting was not applied because this is a narrative, classification-based review rather than a systematic review. Both adult and foetal human data were eligible; interpretations were separated where appropriate.

### 1.6. Risk Grading Criteria (Integrated Approach)

Baseline risk was assigned by proximal plantaris variant geometry (Olewnik Types I–VI; multi-headed/accessory patterns). Risk was upgraded when (1) provoked Doppler US demonstrated reproducible haemodynamic compromise under standardised manoeuvres and/or (2) axial MRI/MRA showed a clear band sign indenting the artery or abutting neural structures. Risk was downgraded when provoked testing was negative and muscle volume was small. The resulting integrated grade (low/moderate/higher) guided conservative vs. surgical decision-making.

## 2. Anatomical Background: Proximal Plantaris Belly Origins (Olewnik Classification)

A six-type framework of proximal PM belly origins was derived from 142 dissected lower limbs, with the plantaris muscle present in 128 (90.1%), on which the classification and prevalence figures are based [13]. Foetal studies corroborate early variability of these attachments, providing a developmental substrate for adult patterns [17].

### 2.1. Definitions and Landmarks (Proximal Focus)

Here, “proximal origin” denotes muscle-belly attachment(s) to the lateral femoral condyle/supracondylar region, the knee joint capsule, the lateral head of the gastrocnemius, and occasionally the iliotibial band (ITB). These structures define the popliteal corridor, within which variant belly bulk and attachment spread can alter relationships with adjacent neurovascular structures [1].

### 2.2. Olewnik Classification (Types I–VI; Adult Cadavers)

**Figure 1:**
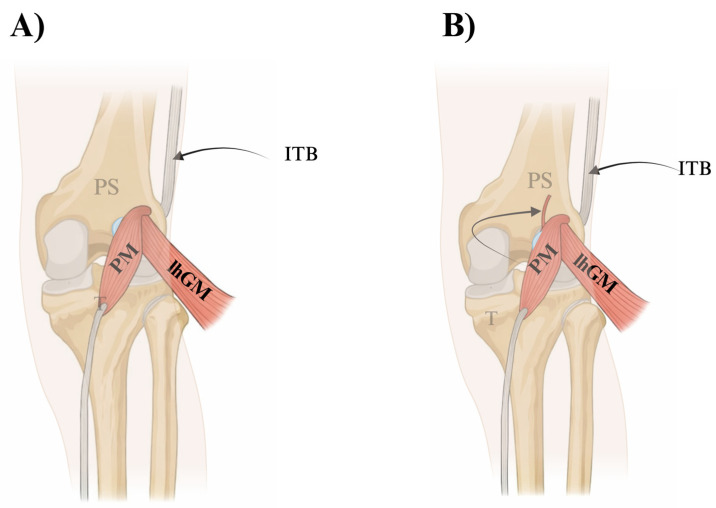
Proximal plantaris muscle (PM) origin variants—Olewnik Type I. (**A**) Type IA: the PM arises from the lateral femoral condyle and knee joint capsule, with fibres blending with the lateral head of the gastrocnemius (lhGM). (**B**) Type IB: as in Type IA, but with an additional posterior footprint on the popliteal surface of the femur (arrow), broadening the proximal attachment. Abbreviations: PM—plantaris muscle; PS—plantaris tendon; ITB—iliotibial band; lhGM—lateral head of gastrocnemius; T—tibia.

**Figure 2:**
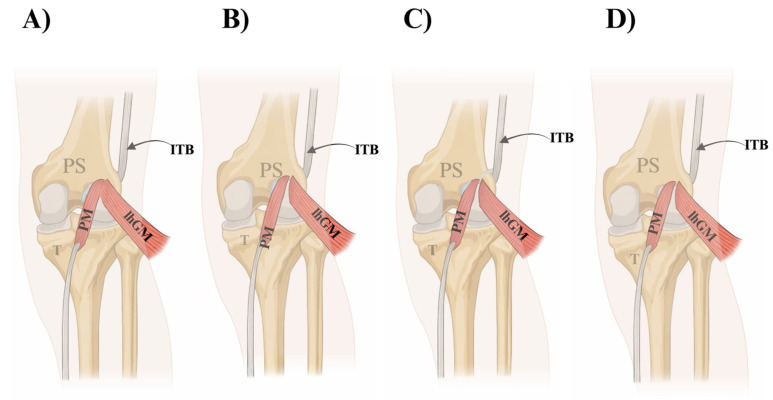
Schematic illustration of plantaris muscle (PM) insertion patterns according to the Olewnik classification (Types II–V) in relation to the lateral knee structures. (**A**) Type II (25.0%; 32/128): the plantaris tendon inserts into the knee capsule and the lateral head of the gastrocnemius muscle (lGCM), maintaining an indirect relationship with the lateral femoral condyle. (**B**) Type III (10.15%; 13/128): the plantaris tendon inserts into the lateral femoral condyle and the knee capsule. (**C**) Type IV (6.25%; 8/128): the plantaris tendon inserts into the lateral femoral condyle, knee capsule, and the iliotibial band (ITB). (**D**) Type V (8.6%; 11/128): the plantaris tendon inserts exclusively into the lateral femoral condyle. Abbreviations: PM—plantaris muscle; PS—plantaris tendon; ITB—iliotibial band; lGCM—lateral head of the gastrocnemius muscle; T—tibia.

**Figure 3:**
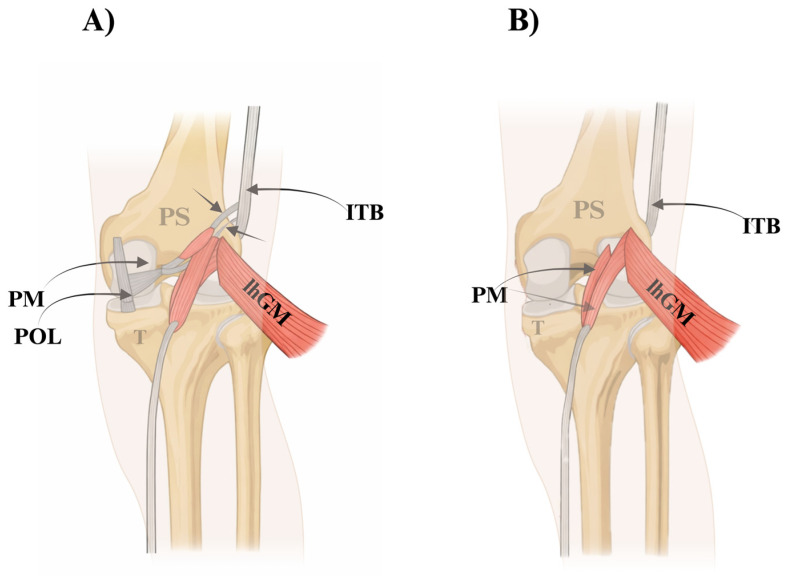
Schematic illustration of plantaris muscle (PM) insertion patterns according to the Olewnik classification—Type VI, representing rare or heterogeneous variants not encompassed by Types I–V (1.6%; 2/128). (**A**) Variant demonstrating a complex relationship of the plantaris tendon (PS) with the posterolateral knee capsule, in proximity to the iliotibial band (ITB) and popliteal ligament complex (POL). (**B**) Variant showing an atypical insertion of the plantaris tendon in the region of the lateral femoral condyle and knee capsule, with close association to the lateral head of the gastrocnemius muscle (lGCM). Abbreviations: PM—plantaris muscle; PS—plantaris tendon; ITB—iliotibial band; lGCM—lateral head of gastrocnemius muscle; POL—popliteal ligament complex; T—tibia.

- Type I (48.4%): two subtypes.
- IA (39.8%; 51/128): origin from the lateral head of gastrocnemius, lateral femoral condyle, and knee capsule—Figure 1A.
- IB (8.6%; 11/128): as in IA plus attachment to the popliteal surface of the femur—Figure 1B.

- **Type II (25.0%; 32/128):** knee capsule and lateral head of gastrocnemius, with **indirect relation** to the lateral femoral condyle—Figure 2A.
- **Type III (10.15%; 13/128):** lateral femoral condyle and knee capsule—Figure 2B.
- **Type IV (6.25%; 8/128):** lateral femoral condyle, knee capsule, and ITB—Figure 2C.
- **Type V (8.6%; 11/128):** lateral femoral condyle only—Figure 2D.

- **Type VI (1.6%; 2/128):** atypical/heterogeneous patterns not encompassed by Types I–V—Figure 3.

To facilitate application of the proximal anatomy, Table 1 summarises plantaris belly origin types according to the six-type framework derived from 142 dissections, with prevalence calculated among limbs with present plantaris (n = 128). For each variant, proximal attachments, prevalence, key relations in the popliteal corridor, and concise corridor implications are listed. Special multi-head and split-belly configurations are case-based (no population percentages) and, in the 2020 framework, fall under Type VI (rare cases).

### 2.3. Special Proximal Variants That Expand Belly Bulk

**Duplication (double PM).** Coexistence of a main belly (e.g., lateral femoral condyle + capsule ± ITB) and an accessory belly (often ITB-related) increases proximal muscle volume [22,23].

**Bifurcated (split) belly.** Two-headed configuration with lateral fibres arising from the lateral head of gastrocnemius and medial fibres from the knee capsule (deep to gastrocnemius); the proximal expansion may create fibromuscular channels [15].

**Extended multi-head spectrum.** Reports include bicipital origins [7] and tri- to four-headed bellies [6,9,15,17], indicating that added heads/slips constitute a proximate mechanism for increased local bulk near neurovascular structures.

**Accessory proximal tendinous slips.** Additional slips contributing to the proximal attachment complex may redirect the immediate corridor without distal mechanics [10] (Nayak et al., 2009). Note: In the original 2020 study [13], such rare multi-head/split configurations are encompassed within **Type VI (rare cases)**.

### 2.4. Rationale for Compression Relevance

Variant belly mass and attachment geometry can narrow or reshape the popliteal corridor, predisposing the muscle to vascular (PAES) or neural conflicts, sometimes in conjunction with neighbouring muscles [19,20]. Rare proximal relationships of adjacent structures have also been implicated in neuropathic symptoms [8,18,21]. These observations motivate a belly-first, type-specific risk matrix in the next Section.

### 2.5. Mechanistic Considerations (Proximal Corridor Only)

1. **Mass effect/corridor crowding.** Added heads, broader footprints, or lateral soft-tissue linkages enlarge belly bulk abutting neurovascular structures [2,3].
2. **Fibromuscular “tunnel” formation.** Geometry at the capsule–gastrocnemius interface can create clefts where vessels or nerves are tethered during knee motion [13,19,20].
3. **Vector redirection of the proximal tendon segment.** The immediately adjacent proximal tendon can be deflected by variant bellies or accessory slips, producing neural contact (e.g., interposition near the tibial nerve (TN)) [8,18,21].

Clinical Box 1 distils variant-indexed red flags and reporting cues pertinent to the proximal corridor. It is intended to complement Table 1 for radiological reporting and pre-operative planning.

Box 1 **Proximal plantaris variants (imaging and surgical cues).**

**When to suspect a proximal plantaris contribution**

- Exertional calf pain/claudication in young, athletic patients ± normal resting pulses.
- Posterior knee pain or paraesthesia provoked by flexion (runner/cyclist profile).
- Prior negative Achilles-focused work-ups despite persistent symptoms.

**Variants with higher theoretical risk (see Table 1)**

- Type IB (broader posterior spread).
- Type II (capsule–gastrocnemius cleft).
- Type IV (iliotibial band linkage).
- Multi-headed forms (duplication/bifurcation/≥3 heads).

**Imaging read-outs to include (proximal corridor)**

- Asymmetric belly bulk abutting artery/nerve; state attachments (LFC, capsule, ITB) and head count.
- Look for a capsule–gastrocnemius cleft/tunnel and proximal tendon redirection (immediate segment only).
- Recommend dynamic Duplex/Doppler (provocation) when anatomy suggests potential compression.

**Reporting phrase (template)**

- “Proximal plantaris variant [Type …]: attachments to [LFC/capsule/ITB], with increased belly bulk adjacent to [popliteal artery/tibial or CPN]. Findings may narrow the popliteal corridor; correlate with exertional symptoms and dynamic vascular testing.”

**Surgical planning cues**

- Expect crowding/tunnels in Type II and multi-head patterns; plan decompression along the cleft.
- In lateralised variants (Type IV, multi-head with ITB linkage), anticipate lateral corridor reshaping and verify the CPN window.

## 3. Vascular Compression Syndromes

### 3.1. Mechanisms of PAES: Classical vs. Functional

Classical (anatomical) PAES—fixed morphologic drivers: In classical PAES, fixed structural anomalies in the popliteal fossa create a reproducible site of arterial compression. For the PM, the relevant drivers are variant proximal bellies (additional heads, broadened footprints, lateral soft-tissue linkages) and, where unavoidable, the immediately adjacent proximal tendon segment [19,20,21]. Two recurring mechanisms are supported by the literature:

Mass effect/fibromuscular sling: Aberrant or multi-headed PM bellies alone or in combination with soleus or the lateral head of gastrocnemius narrow the popliteal corridor and form a muscular/tendinous sling around the artery [1,3,19,20].

Corridor reshaping by variant attachments. Broader or shifted proximal origins (e.g., posterior spread to the popliteal surface or lateral linkage to the ITB/Kaplan fibres) alter the artery–muscle relationship and predispose the muscle to fixed compression points [9,15,21].

Special proximal variants: Double, bifurcated, bicipital, and three-/four-headed bellies intensify these effects by increasing local bulk or forming tunnel-like clefts [6,7,9,15,17,22,23,24]. Rare proximal tendon courses immediately adjacent to the belly may also participate in fixed constriction [21]. Illustrative cases: Aberrant plantaris causing anatomical PAES [19]; neurovascular compression by soleus and plantaris together [5].

#### Functional (Dynamic) PAES—Load-Dependent Drivers

In functional PAES, no obligate structural anomaly is required; instead, muscle hypertrophy or exertional co-contraction in otherwise normal anatomy produces dynamic arterial compression, typically during plantarflexion or deep knee flexion [2,3,5]. Within a plantaris-centric view, the following can be noted:

High-volume bellies (including multi-head variants) can transiently crowd the artery during athletic tasks, amplifying contact forces without a discrete anomalous band [2,5].

Capsule–gastrocnemius interface dynamics: At the capsulo–gastrocnemius junction (e.g., Type II), contraction may tighten a physiologic cleft around the artery, converting a normal corridor into a functional choke point [13].

### 3.2. Variant-Specific Risk

Approach: Risk grades integrate belly geometry (mass effect, attachment spread) and the immediately adjacent proximal tendon corridor (Section 2; Table 1), synthesising anatomical data with PAES case evidence [13,19,20].

Risk summary: Variants with compact lateral footprints—Type I (IA) and Type V—generally confer low baseline crowding; IB adds posterior spread and may escalate risk when hypertrophic [13]. Capsule-related configurations—Type II (capsulo–gastrocnemius junction) and Type III (condylar–capsular proximity)—are best regarded as moderate, given their propensity to form dynamic clefts that narrow with contraction or deep flexion [13,19]. Lateralised linkage—Type IV with ITB/Kaplan fibres expands the lateral footprint and is plausibly potentially higher-risk in view of corridor reshaping and band-like contacts [9,13,15]. Type VI is heterogeneous and remains variable (pattern-dependent) [13]. Accessory head configurations—duplication, bifurcation, and ≥3-headed bellies increase local bulk and can produce tunnel-like channels, aligning with high risk for anatomical PAES [9,15,22,23,24]. Variant-indexed grades are collated in Table 2, while a concise diagnostic workflow is provided in Clinical Box 2.

Box 2 **Work-up for suspected PAES (plantaris-centric).**

**Work-up checklist**

* *

**Clinical triggers**

- Young/athletic patient with exertional calf pain/claudication and normal resting pulses.
- Reproducible symptoms with plantarflexion or deep knee flexion; consider bilateral involvement.

**First-line test**

- Dynamic Duplex/Doppler at the popliteal level with provocation (plantarflexion against resistance; deep flexion).
- Look for loss of colour fill/dampened spectral waveform or velocity drop during provocation with recovery at rest.

**MRI/MRA confirmation**

- Axial mapping for a low-signal fibromuscular band/belly contiguous with proximal plantaris attachments (±capsule, ±iliotibial band/Kaplan fibres).
- Identify variant type (attachments, head count) explaining mass effect; seek a “band sign”.

**High-risk pointers (for surgical planning)**

- Type IV (iliotibial band/Kaplan linkage), duplication/bifurcation, and ≥3-headed bellies (lateral corridor reshaping or tunnel formation).
- Capsule–gastrocnemius cleft (Type II) suggesting dynamic narrowing; correlate with Doppler.

**Avoid common pitfalls**

- Static-only Doppler acquisitions; distal-only imaging focus (Achilles/plantaris tendinopathy).

### 3.3. Clinical Presentation (Young Patients Without Atherosclerosis)

PAES related to proximal plantaris variants typically affects adolescents or young adults, often athletes who develop exertional calf pain/claudication in the absence of atherosclerotic risk factors and with a normal resting vascular examination [2,5,19,20]. Symptoms are load-dependent (running, plantarflexion-dominant effort, deep knee flexion) and remit with rest; provocation may elicit coldness, pallor or fatigue of the foot and can be unilateral or bilateral [19,20]. When neural contact coexists (belly or immediate proximal tendon segment), paraesthesia or numbness may occur in TN/CPN/sural distributions [8,18,21]. Misclassification as chronic exertional compartment syndrome is common when vascular testing is static only, and distal-focused imaging (Achilles/plantaris tendinopathy) may distract from a proximal mechanism [5,25,26,27]. For a stepwise work-up aligned to these features, see Clinical Box 2; risk stratification used in this review is summarised in Table 2.

### 3.4. Imaging Markers: Dynamic Doppler and the MRI “Band Sign” (Proximal Corridor Only)

#### 3.4.1. Provoked Doppler US (First-Line)

The muscle demonstrates physiologic compromise under provocation. We acquired baseline and provoked scans (active plantarflexion against resistance; deep knee flexion; optional forced dorsiflexion with the knee extended). Positive markers include the following: loss/reduction in colour fill, dampened/flattened spectral waveform or velocity drop during provocation with recovery at rest, co-localised to an enlarged/variant PM belly or the immediate proximal tendon segment, ideally with symptom reproduction [5,19,20]. 

#### 3.4.2. MRI/MRA “Band Sign” (Anatomical Confirmation/Mapping)

A low-signal fibromuscular band or belly contiguous with the proximal PM attachment complex (±capsule, ±ITB/Kaplan fibres) that contacts, indents, or encircles the popliteal artery can be seen [13,19,20]. Key readouts (axial emphasis) include the following: culprit band/belly; arterial indentation/tapering (±post-stenotic calibre change); variant mapping (attachments, head count) explaining mass effect; and immediate proximal tendon slip if present. Positional MRI/MRA (neutral vs. provoked) can help separate classical from functional contributions.

## 4. Neural Compression Syndromes

### 4.1. Nerves Affected: Tibial, Common Peroneal, Sural

The TN is the principal nerve at risk in the popliteal fossa. Variant proximal PM bellies (additional heads, broadened footprints) or, when unavoidable, the immediately adjacent proximal tendon segment can alter the corridor and contact the nerve. Direct TN conflict has been documented, including interposition of the plantaris tendon between the TN and a tibial branch [28], as well as rare relations to the popliteal neurovascular bundle [21]. CPN—Lateral spread of the proximal origin (e.g., linkage to the ITB/Kaplan fibres), and multi-headed bellies (Type IV; multi-head spectrum) narrow the lateral window [8,9,13,15].

Sural nerve: Fascial bridges or accessory slips/bellies, particularly in bifurcated or duplicated configurations, may form tunnel-like spaces and predispose the muscle to entrapment [21,22,23].

Variant-indexed neural risk is summarised in Table 3, and a concise clinical workflow appears in Clinical Box 3.

Box 3 **Neural compression in the popliteal fossa (plantaris-centric).**

**Neural entrapment—quick checklist**

* *

**Clinical triggers**

- Young/athletic patient with exertional paraesthesia in TN/CPN/sural distributions ± calf pain.
- Symptoms provoked by plantarflexion or deep knee flexion; resolution at rest; possible bilateral involvement.

**Focused examination**

- Reproduce symptoms with resisted plantarflexion or sustained dorsiflexion (knee extended).
- Palpate popliteal fossa for focal tenderness or a “mass-like” multibelly contour; compare sides.
- Screen for dynamic pulse changes (coexistent vascular component).

**Imaging checklist (US/MRI)**

- Ultrasound: dynamic assessment for belly–nerve contact; trace continuity to recognised proximal attachments.
- MRI (axial emphasis): identify variant type (attachments, head count), capsular clefts (Type II/III), lateral linkage to ITB/Kaplan fibres (Type IV).
- Look for tunnels/loop-around courses in duplication/bifurcation/≥3 heads; avoid “mass” labelling—confirm muscle-like signal and fibre continuity.

**Reporting phrase (template)**

- “Variant plantaris belly [Type …/duplication/bifurcation/≥3 heads] with [capsular cleft/lateral linkage/accessory slip], in contact with the [TN/CPN/sural]. Findings align with exertional paraesthesias; consider dynamic correlation.”

**Differential/pitfalls**

- Lumbar radiculopathy, chronic exertional compartment syndrome, ganglion/cyst.
- Variant-unaware reads: mislabelling multi-headed bellies as cyst/tumour; distal-only focus (Achilles).

**When to consider referral**

- High-risk anatomy (Type IV, duplication/bifurcation/≥3 heads) or progressive neurological deficits despite conservative management.

### 4.2. Mechanisms: Tethering, Dynamic Compression, Fascial Tunnels

(i) Tethering by variant bellies/accessory slips.

Type II/III variants place plantaris fibres across capsular planes that can tether TN during knee motion; Type IV and three-headed variants with Kaplan linkage reduce the CPN corridor. Accessory proximal slips may redirect the immediate tendon segment toward nerves, including true interposition [9,10,18,21].

(ii) Dynamic compression during plantarflexion.

Hypertrophic or multi-headed bellies can transiently crowd neural structures during active plantarflexion or deep knee flexion; this functional component often coexists with anatomical risk [5,13,19,20].

(iii) Fascial entrapment and fibromuscular tunnels.

Duplication/bifurcation may create tunnels with regional fascia (e.g., popliteal fascia, oblique popliteal ligament) for sural or peroneal components [8,21,22,23].

For symptom triage and exam/imaging checkpoints, see Clinical Box 3.

### 4.3. Variant-Specific Neural Entrapment Risk

Approach—Grades reflect contact/tethering potential of the proximal belly and, only when unavoidable, the immediate proximal tendon segment toward TN/CPN/sural within the popliteal corridor.

Risk summary—Low: Type I (IA) and Type V (volume-dependent only); Moderate: Type II/III (capsular clefts, TN tethering); Moderate–High: Type IV (ITB/Kaplan linkage, narrowed CPN window, deeper TN adjacency); High: duplication/bifurcation/≥3 heads (tunnels/loop-around courses) [3,9,13,15,17,18,22,23].

Detailed mapping and representative sources are collated in Table 3.

### 4.4. Clinical Presentation: Calf Pain and Paraesthesias Mimicking Radiculopathy

Adolescents/young adults, often athletes, report exertional calf pain resolving with rest and paraesthesia in TN/CPN/sural distributions, provoked by plantarflexion or deep knee flexion [2,5,19,20]. Variant proximal bellies (multi-head, lateral linkage) may irritate nerves directly or via tunnels; exceptionally, the immediate proximal tendon segment may interpose between TN and a branch [8,18,21]. Features arguing against a spinal origin include reproducibility with local provocation, side-to-side popliteal asymmetry, and dynamic pulse changes when a vascular component coexists [19,20].

Practical triage and phrasing templates are provided in Clinical Box 3.

### 4.5. Imaging Markers: Dual Bellies Misinterpreted as Cyst or Tumour

Pitfall: Duplicated/bifurcated or multi-headed PM bellies can be misread as cyst/soft-tissue tumours, particularly when heads are asymmetric or separated by fascial planes [6,15,17]. Reviews highlight frequent misinterpretation unless the variant is actively sought [2,5]; acute plantaris pathology can mimic other entities [2].

What to look for (belly-first): Continuity with recognised proximal origins (lateral femoral condyle, capsule, lateral head of gastrocnemius, ±ITB/Kaplan) rather than an encapsulated lesion; symmetry/head-count (double, bifurcated, ≥3 heads) with convergence proximally; muscle-like signal with preserved architecture and absence of cystic fluid signal/rim enhancement; and topographic logic (Type IV lateralisation; Type II/III capsular clefts). Align physiology with anatomy: where vascular symptoms coexist, seek a plantaris-related MRI “band sign” at the level of provoked Doppler flow reduction [13]—Section 3.4.

Reporting tip: Name the variant (e.g., “bifurcated plantaris belly”, “three-headed plantaris with Kaplan linkage”) and state its relationship to TN/CPN/sural and/or the popliteal artery [13].

## 5. Radiological and Surgical Detection

### 5.1. MRI and Ultrasound Features by Variant (Proximal Corridor Only)

Modality roles: Dynamic Duplex/Doppler ultrasound demonstrates physiology (loss of colour fill, flattened spectra or velocity drop during provocation, active plantarflexion or deep knee flexion with recovery at rest, and symptom reproduction), whereas MRI/MRA demonstrates anatomy by depicting the axial “band sign”, a low-signal fibromuscular band/belly contiguous with the proximal plantaris origin complex (capsule/lateral femoral condyle [LFC]/lateral head of gastrocnemius [GM-LH] ± ITB/Kaplan fibres) that contacts, indents, or encircles the popliteal artery and delineates head count and attachment spread [2,5,13,19,20].

#### 5.1.1. Variant-Level Recognition

- Type I–IA (GM-LH + LFC + capsule; low risk). US: often normal or only minor dynamic change with strong provocation. MRI: canonical lateral/posterolateral belly; no discrete culprit band unless hypertrophic.
- Type I–IB (IA + popliteal surface; low → higher if bulky). US: subtle posterior lumen narrowing during forceful plantarflexion. MRI: broader posterior footprint; shallow arterial indentation when robust.
- Type II (capsule + GM-LH; moderate; capsular cleft/tethering). US: provoked flow drop at the capsule–GM junction with symptom reproduction. MRI: crescentic low-signal capsular margin adjacent to vessel/nerve; narrow cleft between GM-LH and capsule.
- Type III (LFC + capsule; moderate; condylar–capsular plane). US: dynamic reduction more evident with knee flexion than ankle manoeuvres. MRI: small condylar–capsular window; volume-dependent mass effect.
- Type IV (LFC + capsule + ITB/Kaplan fibres; high vascular, moderate–high neural). US: mixed or fixed pattern; lateral band-like contact on greyscale. MRI: lateralised belly/bridging fibres in continuity with ITB/Kaplan contacting the artery; assess the CPN window laterally.
- Type V (LFC only; low risk). US/MRI: compact lateral belly; no culprit band unless hypertrophic.
- Type VI (atypical; variable). US/MRI: case-by-case mapping; combine dynamic US with targeted axial MRI to define unique corridor geometry.

#### 5.1.2. Multi-Head/Accessory Proximal Variants

- Duplication: US two bellies forming a tunnel-like channel; MRI twin bellies separated by fascia with vessel/nerve straddled/drape.
- Bifurcation: US dual bands around vessel/nerve; MRI two heads (lateral from GM-LH; medial from capsule) converging proximally; tunnel sign.
- Bicipital/≥3-headed spectrum: MRI larger proximal volume and multifocal contact; with Kaplan/ITB linkage expect lateral corridor compromise for CPN.
- Accessory proximal tendinous slip: US linear echogenic slip adjacent to the belly; MRI low-signal slip contiguous with PM capable of interposing between TN and a branch.

Full imaging workflow and reporting checklist → Clinical Box 4. Variant-by-variant pitfalls and corrective steps → Table 4.

Box 4 **Variant-indexed imaging and surgical checkpoints (proximal corridor).**

**Radiology → Surgery: quick pathway**

* *

**Imaging algorithm**

- Dynamic Duplex/Doppler to prove physiology (active plantarflexion, deep knee flexion; recovery at rest).
- Axial MRI/MRA to prove anatomy: “band sign”, head count, attachments (capsule/LFC/GM-LH ± ITB/Kaplan); consider positional sequences.
- Side-to-side comparison and symptom concordance at the same level.

**Variant-specific quick cues**

- Type I IA/IB: usually no discrete band; IB—posterior indentation only if bulky.
- Type II: capsule–GM cleft; Doppler drop at cleft; crescentic capsular margin on MRI.
- Type III: narrow condylar–capsular window; effects accentuated with knee flexion.
- Type IV: lateral band continuous with ITB/Kaplan; assess CPN window.
- Duplication/bifurcation/≥3 heads: twin/dual/multibelly tunnel; avoid cyst/neoplasm misread.
- Accessory proximal slip: linear low-signal slip potentially interposed with TN/branch.

**Reporting checklist (US/MRI)**

- Name the variant and head count (Type …/duplication/bifurcation/≥3 heads).
- Map attachments (capsule, LFC, GM-LH, ±ITB/Kaplan) and continuity across serial axials.
- State relation to artery and to TN/CPN/sural; level of maximal effect.
- Include dynamic correlation (provocation protocol, response, symptom reproduction).
- Exclude mass/cyst by muscle-like signal and fibre continuity.

**Surgical plan cues**

- Type II/III: dissect along capsule; protect TN posteriorly/deep.
- Type IV: distinguish PM vs. ITB/Kaplan; protect CPN; release lateral band.
- Duplication/bifurcation/≥3 heads: search for tunnels/fascial bridges; decompress narrowest channel; address all contributory heads/slips.
- Accessory slip: targeted release at cross-over with TN/branch; preserve GM/soleus fibres.
- Classical PAES: intraoperative Doppler to confirm decompression.

**Common pitfalls → fixes**

- Static-only US → always provoke; document recovery at rest.
- Mass-like read → prove continuity with PM fibres and muscle-like signal; count heads.
- Under-called dynamic clefts → consider positional MRI/MRA.
- Distal-only focus → ensure proximal corridor is mapped.

**When to escalate**

- High-risk anatomy (Type IV, duplication/bifurcation/≥3 heads) with concordant physiology/symptoms.
- Progressive neurological deficit or signs of limb ischaemia.

### 5.2. Diagnostic Pitfalls

The dominant errors are as follows: (i) Type IV misread as mere ITB thickening (masking by continuity with ITB/Kaplan); (ii) duplication/bifurcation misread as cyst/neoplasm (asymmetric, mass-like heads); (iii) Type II/III under-called on static studies (clefts are dynamic); and (iv) acute plantaris pathology (oedema/rupture) confounding interpretation. Corrections include proving continuity with plantaris fibres across serial axial images and the attachment triad (LFC + capsule + ITB/Kaplan), enforcing provocation during US and, where available, positional MRI/MRA, and aligning semiology with Doppler dynamics and proximal-origin topology.

A structured “pitfall → fix → intraoperative checkpoint” matrix is provided in Table 4.

### 5.3. Surgical Considerations (High-Level, Variant-Directed)

Pre-operative mapping should couple dynamic US (level of physiological compromise) with MRI/MRA (culprit belly/head[s], and relationships to TN/CPN/sural and the popliteal artery). Anticipate capsular clefts/tethering in Types II/III (dissect along capsule; protect TN posteriorly/deep), and a lateral band in Type IV (differentiate plantaris from ITB/Kaplan; protect CPN proximally) should also be noted. For duplication/bifurcation/≥3 heads, search for tunnels and fascial bridges and decompress the narrowest channel; for an accessory proximal slip, plan targeted release at the crossing with TN/branch. In suspected classical PAES, ensure vascular precautions and confirm decompression intra-operatively (e.g., Doppler) after releasing the culprit band/belly.

Concise, variant-indexed checkpoints bridging imaging and the operating theatre → Clinical Box 4; detailed “dos and don’ts” by variant → Table 4.

## 6. Proposed Risk Framework

### 6.1. Integrated View: Vascular + Neural Compression Risk (Proximal Corridor Only)

We integrate variant anatomy of the proximal plantaris belly with provoked physiology to yield a single, action-oriented grade per limb. Two axes are reconciled:

- Vascular axis (PAES): Fixed fibromuscular bands/bellies or corridor crowding underlie classical PAES; hypertrophy without an obligate anomaly drives functional PAES [5,19,20].
- Neural axis (TN/CPN/sural): Capsular clefts, lateral linkage (ITB/Kaplan), and multi-head configurations favour tethering/tunnel effects; exceptionally, the immediately adjacent proximal tendon segment contributes when interposed with nerves [8,13,18,21].

#### 6.1.1. Rule Set for an Integrated Grade

Start from the higher of the variant’s vascular or neural baseline grade (see Table 2 and Table 3), then apply the following:

- +1 tier if either (a) MRI shows a band sign with vessel/nerve deformation or (b) dynamic Duplex shows reproducible luminal compromise with symptom reproduction.
- +1 tier for multi-head bellies (double/bifurcated/≥3 heads) or Type IV with proven ITB/Kaplan continuity.
−1 tier if rigorous provocation elicits no dynamic change and the belly is physiologic/small in volume.

#### 6.1.2. Baseline by Variant (Triage Anchor)

- Low: Type I (esp. IA) and V; escalate in IB only if bulky/posteriorly spread.
- Moderate: Types II/III—capsular cleft/condylar–capsular window.
- High: Type IV (ITB/Kaplan linkage) and multi-head variants (double/bifurcated/≥3 heads)
- Variable: Type VI—pattern-dependent.

Red flags (override): Any band sign contacting the artery (classical PAES) or documented interposition with the TN warrants at least a moderate–high integrated grade regardless of baseline.

### 6.2. Stepwise Clinical Approach

#### 6.2.1. Step 1—Identify and Name the Variant (Imaging)

Use dynamic Doppler at the popliteal level (baseline → active plantarflexion/deep knee flexion → recovery) to document haemodynamic change and symptom reproduction; axial MRI/MRA for the band sign, head count (double/bifurcated/≥3), capsular clefts (Types II/III), and ITB/Kaplan linkage (Type IV); and map relations to artery and TN/CPN/sural. Only when unavoidable, confirm a contributing proximal tendon slip.

Guard against Type IV ↔ ITB thickening and multi-head ↔ cyst/tumour misreads using serial axial continuity and muscle-like signal.

#### 6.2.2. Step 2—Correlate with Symptoms (Axis Weights)

Vascular: exertional calf claudication with dynamic pulse changes in young, non-atherosclerotic patients.

Neural: distribution-specific paraesthesia/numbness (TN/CPN/sural), often plantarflexion-provoked and radiculopathy-like yet localisable to the popliteal corridor. Align the site of maximal imaging abnormality with the provoking manoeuvre; discordance should downgrade probability.

#### 6.2.3. Step 3—Apply the Modifiers (Then Plan)

Use Table 2 and Table 3 for the baseline, apply §6.1 modifiers, and plan accordingly:

Low (Type I/V): training-load optimisation; repeat dynamic US if symptoms evolve; MRI only with new red flags.

Moderate (Type II/III): confirm functional physiology; consider targeted interventions; in mixed cases, discuss capsular-cleft decompression.

High (Type IV; multi-head): if conservative care fails, proceed to variant-directed surgery—anticipate lateral band/tunnel anatomy; protect CPN and the popliteal artery; address all contributory heads/slips identified on MRI.

Variable (Type VI): tailor to mapped pattern with imaging–symptom concordance.

Documentation checklist. Report the variant name (e.g., Type IV with ITB linkage; bifurcated belly), risk axis (vascular, neural, or both), provocation result (dynamic Doppler), MRI evidence (band sign/head count/linkage), and target of intervention [13,18,19,20].

Table 5 integrates vascular (PAES) and neural (TN/CPN/sural) risk by starting from the higher baseline grade in Table 2 and Table 3 and applying the §6.1 modifiers: +1 tier for a clear MRI “band sign” with vessel/nerve deformation or reproducible dynamic Duplex/Doppler physiology, and +1 tier for multi-head bellies or Type IV with proven ITB/Kaplan continuity; −1 tier when rigorous provocation is negative and the belly volume is physiologically small [2,13,19,20,24]. For a practical workflow bridging imaging and theatre, see Clinical Box 4; common pitfalls and intraoperative checkpoints are summarised in Table 4.

Two brief clinical vignettes illustrate how the integrated risk matrix informs management (see Clinical Box 5 and Box 6).

Box 5 **Clinical vignette—application of the integrated risk matrix.**

**A. Athlete, Type IV (lateral linkage)**

Patient: 19-year-old sprinter with exertional calf claudication.

Variant (anatomy): Type IV (lateral linkage with plantaris–iliotibial band [ITB]/Kaplan fibres), which geometrically narrows the lateral popliteal corridor under load.

Provoked Doppler US (physiology): Reproducible velocity drop during resisted plantarflexion.

MRI/MR angiography (MRA) (anatomy): Axial low-signal band sign contiguous with the plantaris–ITB/Kaplan complex indenting the popliteal artery.

Integrated grade: High (concordant higher-risk variant + reproducible physiological compromise + axial band sign).

Plan: Early multidisciplinary team (MDT) review; targeted lateral band release with common peroneal nerve (CPN) protection.

Key message: Concordant anatomical and physiological signals in a higher-risk variant support early, targeted decompression.

Abbreviations: ITB—iliotibial band; CPN—common peroneal nerve; MDT—multidisciplinary team; MRA—MR angiography.

Box 6 **Clinical vignette—application of the integrated risk matrix.**

**B. Runner, Type II (capsular junction)**

Patient: 27-year-old endurance runner with paraesthesia in the tibial nerve (TN) distribution.

Variant (anatomy): Type II (capsular-junction pattern), baseline moderate risk.

Provoked Doppler US (physiology): Mild, non-reproducible change.

MRI/MR angiography (MRA) (anatomy): Narrow capsule–gastrocnemius cleft without arterial indentation; no axial band sign.

Integrated grade: Moderate → Low (no consistent physiological or anatomical conflict).

Plan: Conservative management and workload modulation; re-test with standardised provocation if symptoms progress.

Key message: Inconsistent physiological and anatomical signals in a moderate-risk variant favour conservative management and reassessment.

Abbreviations: TN—tibial nerve; MRA—MR angiography.

## 7. Future Directions

### 7.1. Prospective Imaging Studies Correlating PM Variants with Entrapment Syndromes

Multicentre, prospective cohorts are needed to link named proximal-belly variants (per the Olewnik classification) with adjudicated clinical outcomes in vascular and neural entrapment. A pre-registered protocol should (i) assign a variant type on axial MRI; (ii) capture dynamic Duplex/Doppler physiology under standardised provocation; and (iii) record endpoints (classical/functional PAES, distribution-specific neuropathy, need for decompression) [2,5,13,19,20]. Incorporating developmental data (foetal morphology) could clarify which adult patterns reflect congenital templates with later clinical expression [17].

### 7.2. Dynamic Imaging Protocols Under Load

Standardised provocation protocols should be defined and shared across centres so that physiology (Duplex/Doppler) and anatomy (MRI/MRA) are reproducible: fixed ankle/knee positions, side-to-side comparison, and real-time symptom documentation [2,5,19,20]. MRI checklists should mandate axial head counting (single vs. double/bifurcated vs. ≥3), capsular cleft assessment (Types II/III), and ITB/Kaplan continuity (Type IV) to reduce misreads (e.g., ITB thickening, mass-like multi-head bellies) [9,13,17]. Confounders, e.g., acute PM injury on MRI, should be explicitly ruled out [2].

### 7.3. Registry-Based Documentation of Rare Variants (Double, Bifurcated)

A curated Plantaris Variant and Entrapment Registry should capture the following: variant label (Type I–VI; double; bifurcated; bicipital; ≥3-/4-headed; accessory proximal slip), linkages (ITB/Kaplan), dynamic findings, implicated axis (vascular, neural, or both), treatment, and outcomes. Case-based evidence is rich but fragmented; a registry would aggregate patterns such as duplication/bifurcation [22,23,24], three-/four-headed and Kaplan-linked bellies [6,9,15,17], bicipital origins [7], accessory proximal slips [10], nerve interposition [28], anatomical PAES due to aberrant plantaris [19], and rare neurovascular relations in the popliteal fossa [8,21].

### 7.4. Interdisciplinary Collaboration Across Orthopaedics, Vascular Surgery, Neurology, Radiology

We recommend routine variant naming in imaging reports, joint read-outs (radiology–orthopaedics–vascular–neurology), and pre-operative conferences for high-risk patterns (Type IV; multi-head; accessory slips). A shared lexicon and variant-directed algorithms (dynamic Duplex/Doppler first; targeted axial MRI; operative mapping) should reduce under-/over-calling and improve selection for decompression [5,20]. Analogous decision pathways are advocated in other neurovascular districts, where early multidisciplinary planning and nerve-sparing strategies improve safety and outcomes [28]. Consolidated reviews highlight the clinical value of recognising plantaris variability; formalising collaboration is the next step [16].

### 7.5. Limitations

This narrative, classification-centred review synthesises heterogeneous sources; most clinical signals derive from Level IV evidence (case reports/series), with potential selection and publication biases. Imaging protocols and provocation manoeuvres for provoked Doppler US are non-standard across studies, and definitions of the axial “band sign” and plantaris variant boundaries vary, limiting comparability. We restricted the search to English-language publications and did not contact authors for missing data; adult and foetal anatomical data were both included, increasing biological heterogeneity. Distal tendon-only variants were excluded by design. No quantitative validation or inter-rater reliability testing of the proposed risk grades was performed, and the framework has not yet been externally validated, although we sought to mitigate citation bias by incorporating classical PAES taxonomies (e.g., Whelan/Rich, Sinha) and independent imaging series.

## 8. Conclusions

Clinical salience: Proximal-belly variability of the plantaris is unlikely to be merely incidental; lateral linkage patterns and multi-headed configurations may contribute to both vascular and neural entrapment, whereas canonical or compact footprints are generally associated with low risk unless hypertrophic. In clinical practice, recognising variants considered higher-risk and confirming concordant physiological and anatomical signals should prompt early multidisciplinary review and consideration of targeted decompression; discordant or absent signals favour conservative management with reassessment under standardised provocation.

Classification-led practice: Systematic, variant-labelled recognition on imaging, using provoked Doppler US to demonstrate physiological compromise and axial MRI/MR angiography to map anatomy may improve diagnostic confidence and inform surgical planning on capsular clefts, lateral bands, and multi-head tunnels that may warrant targeted release.

Integrated risk pathway: Applying the proposed framework baseline by variant, modified by dynamic physiology and MRI band sign may offer a consistent route from recognition to intervention, helping clinicians distinguish incidental findings. Given that most available clinical data are Level IV (case reports/series), these conclusions are inferential and should be applied with clinical judgement and shared decision-making.

## Figures and Tables

**Table 1:** Proximal plantaris variants—origin, prevalence, and proximal corridor implications (belly-focused). This table summarises the proximal belly origin types of the plantaris muscle (PM) following the six-type framework. Percentages are calculated among limbs with present PM (n = 128 of 142). Entries include proximal relations and inferred implications for the popliteal corridor; special multi-head/split variants are case-based and reported without population prevalence.

Variant (Adult)	Proximal Origin (Attachments)	Prevalence in 128 Limbs	Proximal Anatomical Relations (Belly-Centric)	Corridor Implications (Popliteal Fossa)	Key References
Type I—IA	Lateral head of gastrocnemius (GM-LH) + lateral femoral condyle (LFC) + knee joint capsule	51 (39.8%)	Lateral/posterolateral footprint; belly adjacent to capsule and GM-LH	Baseline configuration; mass effect size-dependent	Olewnik et al., 2020
Type I—IB	As Type I—IA plus popliteal surface of the femur	11 (8.6%)	Broader posterior footprint	Slight corridor narrowing if robust	Olewnik et al., 2020
Type II	Knee joint capsule + GM-LH; indirect relation to LFC	32 (25.0%)	Capsulo–gastrocnemius junction	Possible fibromuscular window at capsule–GM interface	Olewnik et al., 2020
Type III	LFC + knee joint capsule	13 (10.15%)	Lateral femoral–capsular complex	Generally baseline; volume-modulated	Olewnik et al., 2020
Type IV	LFC + knee joint capsule + iliotibial band (ITB)	8 (6.25%)	Lateral soft-tissue linkage via ITB	Lateral spread; potential corridor reshaping	Olewnik et al., 2020
Type V	LFC only	11 (8.6%)	Compact lateral footprint	Typically low mass effect	Olewnik et al., 2020
Type VI	Atypical/heterogeneous patterns	2 (1.6%)	Variable	Pattern-dependent	Olewnik et al., 2020
Double plantaris (duplication)	Main belly: LFC + capsule ± ITB; accessory belly commonly linked to ITB	—(case reports)	Two discrete bellies increase local bulk	Higher likelihood of crowding or tunnel formation	Rana et al., 2006; Kwinter et al., 2010
Bifurcated (split) plantaris	Two heads: lateral from GM-LH; medial from knee capsule (deep to GM)	—(case reports)	Split belly enlarges proximal cross-section	Potential channelling or redirection	Smedra et al.
Bicipital origin	Two-headed proximal attachment with distinct footprints	—(case reports)	Increased attachment spread	Increased mass effect; variant-dependent	Heo et al., 2021
Three-headed plantaris	Three discrete proximal heads	—(case reports)	Marked belly bulk near neurovascular structures	Elevated crowding risk	Triantafyllou et al., 2025; Maslanka et al., 2024
Three-headed plantaris (Kaplan fibres)	Three heads with lateral linkage to Kaplan fibres/ITB	—(case report)	Lateral expansion toward ITB/Kaplan fibres	Lateral corridor reshaping; variant-specific	Maslanka et al., 2024
Four-headed plantaris	Four discrete proximal heads	—(case reports)	Very large proximal muscle volume	High potential for corridor narrowing	Zielińska et al., 2024
Accessory proximal tendinous slip	Additional proximal tendinous slip contributing to attachment complex	—(case reports)	Alters immediate proximal corridor geometry	Possible redirection toward neurovascular structures	Nayak et al., 2009

Footnote: Special multi-head and split-belly configurations belong to Type VI (rare cases) in the 2020 framework (Olewnik et al., 2020).

**Table 2:** Plantaris variants and vascular entrapment risk (proximal belly focus). Risk reflects popliteal corridor crowding/tunnel formation by the belly and, when unavoidable, the immediately adjacent proximal tendon segment. Abbreviations as in Table 1.

Variant (Proximal Belly)	Vascular Entrapment Risk	Primary Anatomical Driver (Belly-Focused)	Typical Mechanism Signature	Imaging Cues (Doppler/MRI)	Representative Sources
**Type I—IA**	Low	Canonical GM-LH + LFC + capsule footprint; limited posterior spread	Functional only if hypertrophic	Mild/negative dynamics; no discrete band	Olewnik et al., 2020; Kurtys et al., 2021; Gonera et al., 2021
**Type I—IB**	Low (↑ if robust)	As IA plus popliteal surface—broader posterior footprint	Mixed; posterior adjacency may permit fixed contact	Subtle posterior band/contact if bulky	Olewnik et al., 2020; Turnipseed & Pozniak, 1992
**Type II**	Moderate	Capsulo–GM junction; potential cleft/tethering	Functional or mixed	Provoked flow drop at capsule–GM level; capsular band	Olewnik et al., 2020; Kwon et al., 2015
**Type III**	Moderate	LFC + capsule (condylar–capsular proximity)	Functional (volume-dependent)	Dynamic reduction; subtle interface band	Olewnik et al., 2020; Kurtys et al., 2021
**Type IV**	High	Added lateral linkage (ITB/Kaplan complex) enlarges lateral footprint	Classical fixed band more likely	Distinct lateral band; corridor reshaping	Olewnik et al., 2020; Maślanka et al., 2024; Triantafyllou et al., 2025
**Type V**	Low	Compact, condylar-only origin	Functional only if hypertrophic	Typically normal; no discrete band	Olewnik et al., 2020
**Type VI**	Variable	Heterogeneous outliers	Pattern-dependent	Case-by-case	Olewnik et al., 2020
**Double PM (duplication)**	High	Two discrete bellies → bulk/tunnel	Classical	Fixed stenosis where twin bellies straddle artery	Rana et al., 2006; Kwinter et al., 2010
**Bifurcated (split) PM**	High	Two heads forming a split belly	Classical	Two-band appearance; tunnel-like channel	Smędra et al., 2021
**Bicipital origin**	Moderate–High	Two-headed proximal attachment with wider spread	Mixed	Broad band/dual slips near artery	Heo et al., 2021
**Three-headed PM**	High	Marked increase in belly volume (≥3 heads)	Classical	Multiband/bulky contact with artery	Triantafyllou et al., 2025; Maślanka et al., 2024
**Three-headed PM fused with Kaplan fibres**	High	Lateral soft-tissue linkage intensifies lateral mass	Classical	Lateral band contiguous with Kaplan/ITB	Maślanka et al., 2024
**Four-headed PM**	High	Very large proximal muscle volume	Classical	Extensive bulky contact; multiband effect	Zielinska et al., 2024
**Accessory proximal tendinous slip**	Moderate (↑ if crossing artery)	Extra proximal slip can redirect immediate tendon corridor toward vessel	Mixed	Linear low-signal slip contacting artery	Nayak et al., 2009; Kwon et al., 2015

**Table 3:** Plantaris variants and neural compression risk.

Variant (Proximal Belly)	Neural Compression Risk	Nerves Most at Risk	Primary Driver (Belly-Focused)	Typical Mechanism Signature	Imaging Cues (US/MRI)	Representative Sources
**Type I—IA**	Low	TN (rare)	Canonical GM-LH + LFC + capsule footprint; limited spread	Volume-dependent irritation only (athletic hypertrophy)	Usually normal; no focal belly–nerve contact	Olewnik et al., 2020; Kurtys et al., 2021; Gonera et al., 2021
**Type I—IB**	Low (↑ if robust)	TN	Posterior spread toward popliteal surface	Closer TN adjacency; symptoms if bulky	Subtle posterior contact contiguous with PM belly	Olewnik et al., 2020
**Type II**	Moderate	TN	Capsulo–GM junction creates dynamic cleft	Capsular tethering of TN with flexion/plantarflexion	Provocation reproduces paraesthesia; capsular margin on MRI	Olewnik et al., 2020; Das & Vasudeva, 2007; Kwon et al., 2015
**Type III**	Moderate	TN	Condylar–capsular proximity	Functional tethering; volume-dependent crowding	Subtle belly at condylar–capsular plane; dynamic symptoms	Olewnik et al., 2020; Kurtys et al., 2021
**Type IV**	Moderate–High	CPN, TN	Lateral linkage (ITB/Kaplan fibres) enlarges lateral bulk	Lateral window narrowing for CPN; deep TN adjacency	Lateralised belly continuity with ITB/Kaplan; focal contact	Olewnik et al., 2020; Maslanka et al., 2024; Triantafyllou et al., 2025; Kotian et al., 2013
**Type V**	Low	TN (rare)	Compact LFC-only origin	Low baseline; hypertrophy-related	No discrete contact	Olewnik et al., 2020
**Type VI**	Variable	TN/CPN/sural (pattern-dependent)	Heterogeneous outliers	Unpredictable	Case-by-case mapping	Olewnik et al., 2020
**Double plantaris (duplication)**	High	TN/CPN/sural	Two discrete bellies increase bulk; tunnels	Fibromuscular tunnels; loop-around courses	Twin bellies with fascial bridges; nerve draped/tented	Rana et al., 2006; Kwinter et al., 2010
**Bifurcated (split) plantaris**	High	TN/CPN/sural	Two heads (lateral from GM-LH; medial from capsule)	Split-belly tunnel with nerve interposition	Dual bellies converging proximally; focal nerve contact	Smedra et al., 2021
**Bicipital origin**	Moderate–High	TN/CPN	Two distinct proximal footprints widen spread	Mixed tethering + mass effect	Two heads near neurovascular paths	Heo et al., 2021
**Three-headed plantaris**	High	TN/CPN	Marked increase in belly volume (≥3 heads)	Corridor crowding; multi-head contact	Multiple bellies abutting nerve(s)	Triantafyllou et al., 2025; Zielinska et al., 2024; Hawi et al., 2024
**Three-headed plantaris (linked to Kaplan fibres)**	High	CPN	Lateral soft-tissue linkage intensifies lateral mass	Lateral corridor tethering of CPN	Continuity with Kaplan/ITB; nerve indentation	Maslanka et al., 2024
**Four-headed plantaris**	High	TN/CPN	Very large proximal muscle volume	Extensive crowding; multichannel tunnels	Multibelly complex encroaching on nerves	Zielinska et al., 2024
**Accessory proximal tendinous slip**	Moderate–High (↑ if interposed)	TN (± branch)	Extra proximal slip redirects immediate tendon corridor	Interposition between TN and branch; focal neuritis	Linear low-signal slip contiguous with PM belly contacting nerve	Nayak et al., 2009; Das & Vasudeva, 2007; Olewnik et al., 2018

**Table 4:** Radiological pitfalls and intraoperative considerations per variant (proximal belly focus).

Variant	Radiological Pitfalls	How to Avoid/Corrective Imaging	Intraoperative Considerations	Representative Sources
**Type I—IA**	Over-calling minor proximity as entrapment in athletic hypertrophy.	Dynamic Doppler to show absent/trace physiology; confirm no discrete band sign on axial MRI.	Generally low-risk; avoid unnecessary release; confirm no other culprit.	Olewnik et al., 2020; Kurtys et al., 2021; Gonera et al., 2021
**Type I—IB**	Posterior spread overlooked; subtle arterial indentation missed on neutral MRI.	Interrogate posterior footprint on axial MRI; add plantarflexion/knee-flexion provocation in US.	If bulky, limited posterior fibre decompression may be needed; verify with intraoperative Doppler.	Olewnik et al., 2020; Turnipseed & Pozniak, 1992
**Type II**	Under-called on static studies; capsular cleft/tethering appears only dynamically.	Target capsule–GM junction; dynamic US with symptom reproduction; consider positional MRI/MRA.	Careful dissection along the capsule; protect TN posteriorly/deep.	Olewnik et al., 2020; Kwon et al., 2015; Das & Vasudeva, 2007
**Type III**	Flow change misattributed to distal causes; narrow condylar–capsular window not recognised.	Bias provocation to knee flexion during US; thin-slice axial MRI to show the window.	Capsular plane release if confirmed; avoid over-resection of lateral fibres.	Olewnik et al., 2020; Kurtys et al., 2021
**Type IV**	Mistaken for ITB thickening/Kaplan fibres; lateral band not recognised as plantaris.	Prove continuity with PM fibres across serial axials; map LFC + capsule + ITB/Kaplan triad; assess lateral band sign and CPN corridor.	Differentiate PM vs. ITB/Kaplan; protect CPN proximally; confirm decompression with Doppler.	Olewnik et al., 2020; Maslanka et al., 2024; Triantafyllou et al., 2025; Kotian et al., 2013
**Type V**	False positives from physiological contact in lean athletes.	Demonstrate lack of dynamic occlusion on Doppler; no discrete culprit on MRI.	Usually observe; if symptomatic, prioritise search for other variants.	Olewnik et al., 2020
**Type VI**	Pattern-dependent errors; unusual courses mislabelled as a mass.	Full variant mapping with axial MRI + dynamic US; side-to-side comparison.	Plan case-by-case; anticipate atypical planes and multiple contact points.	Olewnik et al., 2020
**Double (duplication)**	Misdiagnosed as neoplasm/cyst; twin bellies appear mass-like.	Confirm muscle-like signal, two discrete bellies, proximal convergence; correlate with dynamic physiology.	Search for tunnels/fascial bridges; decompress the narrowest channel; address both bellies.	Rana et al., 2006; Kwinter et al., 2010
**Bifurcated (split)**	Dual heads read as cystic/solid lesion; tunnel not recognised.	Identify lateral (GM-LH) + medial (capsule) heads on axial MRI; show dual bands on US.	Release at the tunnel; ensure both heads are addressed to prevent recurrence.	Smedra et al., 2021
**Bicipital origin**	Underestimation of spread from two proximal footprints near neurovascular paths.	Count heads on MRI; map each footprint relative to artery/nerve; add dynamic US correlation.	Decompress both heads if contributory; avoid residual slips.	Heo et al., 2021
**Three-headed (± Kaplan linkage)**	Head miscount; Kaplan/ITB linkage missed → CPN risk under-called.	Axial stack to count ≥3 heads; check Kaplan/ITB continuity and CPN window.	Protect CPN; release lateral band and crowded channels.	Triantafyllou et al., 2025; Maslanka et al., 2024
**Four-headed**	Mass-like appearance due to volume and septa; tumour mimic.	Confirm multibelly architecture and muscle signal; dynamic US for physiology.	Expect multichannel tunnels; stepwise decompression with Doppler confirmation.	Zielinska et al., 2024; Hawi et al., 2024
**Accessory proximal tendinous slip**	Slip missed or read as scar; interposition with TN not recognised.	Track low-signal slip from PM belly to crossing; document TN/branch relationship; correlate with provoked symptoms.	Targeted release at cross-over; preserve GM/soleus; check neural gliding.	Nayak et al., 2009; Das & Vasudeva, 2007; Olewnik et al., 2018

**Table 5:** Integrated risk matrix (proximal belly variants → vascular risk → neural risk → clinical notes).

Variant (Proximal Belly)	Vascular Risk (PAES)	Neural Risk (TN/CPN/Sural)	Clinical Notes (Integrated)
**Type I—IA**	Low	Low	Canonical footprint; treat hypertrophy as functional amplifier; expect negative/weak dynamics on Doppler; no discrete MRI “band sign” (Olewnik et al., 2020; Kurtys et al., 2021; Gonera et al., 2021).
**Type I—IB**	Low (↑ if robust)	Low	Posterior spread toward popliteal surface; watch subtle posterior indentation on MRI; escalate only with clear dynamics/symptoms (Olewnik et al., 2020; Turnipseed and Pozniak, 1992).
**Type II**	Moderate	Moderate (TN)	Capsule–GM cleft/tethering; prove physiology with provoked Doppler; “band” along capsular margin on MRI; consider mixed vascular–neural symptoms (Olewnik et al., 2020; Kwon et al., 2015; Das and Vasudeva, 2006).
**Type III**	Moderate	Moderate (TN)	Condylar–capsular window; knee-flexion–biased dynamics; volume-dependent crowding; consider targeted capsular decompression if refractory (Olewnik et al., 2020; Kurtys et al., 2021).
**Type IV (±Kaplan/ITB)**	High	Moderate–High (CPN, ±TN)	Lateralised belly/bridge continuity with ITB/Kaplan; MRI lateral “band sign”, CPN window narrowing; high classical PAES likelihood; protect CPN intraoperatively (Olewnik et al., 2020; Maślanka et al., 2024; Triantafyllou et al., 2025; Kotian et al., 2013).
**Type V**	Low	Low	Compact LFC-only origin; avoid over-calling physiological contact; manage conservatively unless new red flags appear (Olewnik et al., 2020).
**Type VI (atypical)**	Variable	Variable	Pattern-dependent outliers; decide by dynamic Doppler + axial MRI mapping and symptom concordance (Olewnik et al., 2020).
**Double (duplication)**	High	High	Two bellies create tunnel and mass effect; avoid “neoplasm/cyst” misread; decompress narrowest channel; address both bellies (Rana et al., 2006; Kwinter et al., 2010).
**Bifurcated (split)**	High	High	Lateral (GM-LH) + medial (capsule) heads; dual-band/tunnel sign; neural looping plausible; treat as classical entrapment when band sign present (Smędra et al., 2021).
**Bicipital origin**	Moderate–High	Moderate–High	Two proximal footprints widen spread near the neurovascular bundle; map both heads; mixed tethering + mass effect (Heo et al., 2021).
**Three-headed PM**	High	High	Marked proximal bulk; multiband contact on MRI; plan multi-channel decompression (Triantafyllou et al., 2025; Maślanka et al., 2024).
**Three-headed + Kaplan linkage**	High	High (CPN > TN)	Lateral soft-tissue linkage intensifies CPN risk; confirm Kaplan continuity; lateral corridor release is key (Maślanka et al., 2024).
**Four-headed PM**	High	High	Very large volume; multichannel tunnels; avoid tumour mimic by proving muscle architecture (Zielinska et al., 2024; Hawi et al., 2024).
**Accessory proximal tendinous slip**	Moderate (↑ if crossing artery)	Moderate–High (TN)	Track low-signal proximal slip contiguous with the belly; interposition with TN/branch elevates neural risk; target cross-over point (Nayak et al., 2009; Das and Vasudeva, 2006; Olewnik et al., 2018; Kwon et al., 2015).

Colour key: risk cells—Low (green), Moderate (yellow), Moderate–High (orange), High (red), Variable (purple).

## Data Availability

No new data were created or analysed in this study.
